# No Unique Magnocellular Facilitation in Parafoveal Processing: A Combined EEG and Eye Tracking Study

**DOI:** 10.1162/NOL.a.223

**Published:** 2026-03-16

**Authors:** Xin Huang, Brian W. L. Wong, Werner Sommer, Olaf Dimigen, Urs Maurer

**Affiliations:** School of Psychology, Nanjing Normal University, Nanjing, China; Department of Psychology, The Chinese University of Hong Kong, Hong Kong, China; BCBL, Basque Center on Brain, Language and Cognition, Donostia–San Sebastián, Spain; Department of Psychology, Humboldt-Universität zu Berlin, Berlin, Germany; Department of Physics and Life Science Imaging Center, Hong Kong Baptist University, Hong Kong, China; Department of Psychology, Zhejiang Normal University, Jin Hua, China; Faculty of Education, National University of Malaysia, Kuala Lumpur, Malaysia; Department of Experimental Psychology, University of Groningen, Groningen, The Netherlands; Brain and Mind Institute, The Chinese University of Hong Kong, Hong Kong, China

**Keywords:** combined EEG and eye tracking, luminance contrast, magnocellular, parvocellular, spatial frequency, visual word recognition

## Abstract

Rapidly processed magnocellular (M) information may facilitate visual object recognition but its role in reading is unclear. A previous study with Chinese characters and masked foveal primes did not find a unique role of the M system as compared to the parvocellular (P) system in mediating repetition effects. As M cells are better represented in the parafoveal visual field, the present study tested whether the M and P systems contribute differentially to parafoveal processing during reading. We combined EEG recordings and eye tracking to measure parafoveal preview effects in fixation-related potentials, using the boundary paradigm. In two experiments, we contrasted high versus low spatial frequency previews and luminance versus color contrast previews and also included standard previews as a manipulation check. As expected, the N250 component was diminished after valid as compared to invalid normal previews, especially over the left hemisphere. We also obtained left-lateralized preview effects for the N250 component for both M- and P-biased previews in both experiments. In the experiment involving a spatial frequency manipulation, P-biased preview effects tended to be larger than M-biased preview effects over the left hemisphere, but not over the right hemisphere. No interactions with preview validity were found for the luminance contrast manipulation. This null effect was supported by a Bayesian analysis. Taken together, these results indicate that the M pathway does not exclusively mediate the preview effect, even for stimuli presented in the parafovea. Instead, both M- and P-based information appear to contribute to early, left-lateralized neural processes underlying visual word recognition.

## INTRODUCTION

Readers sample information not only from the fovea (the central 2° of the visual field) but also from parafoveal regions (eccentricity of about 1°–5° from the point of fixation) despite rather poor spatial resolution (see review by Schotter et al., 2012). The human visual system consists of anatomically and functionally distinct pathways projecting from the retinal magnocellular (M), parvocellular (P), and koniocellular (K) ganglion cells to the lateral geniculate nucleus of the thalamus and to the visual cortex. The current work intends to investigate the contributions of the M and P pathways to visual word recognition, with an emphasis on parafoveal contributions. Therefore, it is important to differentiate both the processing capacities, as well as the cortical projections of the two visual pathways.

The visual system contains two types of photoreceptors: cones, which are sensitive to color and fine detail in bright light (photopic conditions), and rods, which detect luminance under low-light conditions (mesopic and scotopic, i.e., lower than ~10 cd/m^2^). Under the photopic conditions typical of reading, rod photoreceptors are saturated and functionally inactive, and therefore do not contribute meaningfully to visual perception.

The retinal photoreceptors feed into the ganglion cell pathways described above, with cones providing the primary input to both the M and P pathways under photopic conditions. P cells are abundant in and around the fovea, receive input predominantly from cones through midget ganglion cells, and have small receptive fields (Kaplan, 2004). They are small neurons with low contrast sensitivity, high spatial resolution, low temporal resolution, color sensitivity, and slow conduction velocity. In contrast, M cells are largely absent from the fovea but numerous in the retinal periphery, with larger receptive fields that increase in size toward the periphery (Kaplan, 2013; Yoonessi & Yoonessi, 2011). They are large, rapidly conducting neurons that respond preferentially to luminance contrast at low spatial frequencies and are highly sensitive to temporal change.

Although traditionally described as achromatic and luminance-driven, recent evidence suggests that M cells may also carry weak chromatic signals under certain conditions (Lee & Sun, 2009). Functionally, M projections comprise most of the dorsal “where” system, processing motion and spatial location, while P projections form much of the ventral “what” system, processing object identification (Goodale & Milner, 1992). The P system mediates spatial contrast detection across a wide range of spatial frequencies, whereas the M system is more sensitive to luminance contrast than to spatial frequency changes (Legge, 1978; Skottun, 2015).

Given their spatial distribution, it follows that M and P cells may critically shape parafoveal processing capabilities during reading. The transition from P-cell dominance in the fovea to M-cell dominance in the parafovea fundamentally alters how visual information is processed. In foveal regions, abundant P cells provide the high spatial resolution necessary for precise character recognition. However, as the text extends into parafoveal areas, where M cells dominate, the larger receptive fields and reduced spatial resolution create averaged, less distinct visual signals. This M-cell processing results in fuzzy text perception that impairs accurate symbol identification. Consequently, word recognition accuracy drops sharply beyond the fovea, reaching chance levels around 3° in the parafovea (Bouma, 1973; Rayner & Morrison, 1981). The shift from P-cell to M-cell processing in parafoveal regions presents neurological challenges for speed-reading techniques relying on peripheral vision, as M-cell spatial processing limitations may reduce the accuracy of simultaneous word recognition, particularly when bottom-up visual input is insufficient.

Understanding how the visual system processes information through different neural pathways has fundamental implications for visual object recognition. Stimuli that are designed to bias processing toward M pathways appear to facilitate fast and accurate responses compared to P-biased stimuli (Kveraga et al., 2007). Therefore, Bar (2004) proposed a model of top-down facilitation in visual object recognition as part of a predictive coding framework. According to this model, the M system rapidly projects to the orbitofrontal cortex, where coarse information about the stimulus (the gist) is first extracted. This process facilitates subsequent processing in the inferotemporal cortex, part of the occipitotemporal (OT) pathway, through top-down predictions. This influential model has been primarily developed and tested within the domain of object recognition, leaving open the question of whether similar mechanisms operate also in visual word recognition.

An extension of Bar's model from objects to words is supported by neuroanatomical evidence that the OT region subserves both visual object processing and reading (Dehaene & Cohen, 2011; Price & Devlin, 2011). In particular, the ventral OT region (vOT) is commonly activated during visual word recognition, and damage to this area can lead to reading difficulties (e.g., alexia; Starrfelt et al., 2009). In some neural models, the vOT also serves as an interface, associating bottom-up visual form information important for orthographic processing with top-down higher order linguistic information (Kherif et al., 2011; Nakamura et al., 2005). Because the vOT area is important for both visual object and word processing, the same processes that subserve the recognition of objects may also be involved in the recognition of visual (written) words. Therefore, we propose that Bar's dual-pathway model of predictive coding, originally developed for object recognition, may provide a unifying framework for understanding also visual word recognition. The current study directly tests this theoretical extension by examining whether M-biased and P-biased stimuli differentially affect word recognition processes.

Like visual objects, written words contain spatial frequency and luminance contrast information (Schlaggar & McCandliss, 2007). Low spatial frequencies (LSF) often provide information about general word shape, length, and location (Boden & Giaschi, 2009) whereas high spatial frequencies (HSF) typically provide fine-grained characteristics, such as lines and edges, which are critical for letter recognition (Majaj et al., 2002) and, hence, reading (Beckmann et al., 1991; Majaj et al., 2002). However, the role of LSF information in visual word recognition cannot be ignored. Some studies have found that word shapes and, by extension, perhaps LSF information, can support word identification. For example, in isolation, the outer features of a word provide more relevant information for lexical access than the inner features (Beech & Mayall, 2005). Furthermore, letters can still be recognized correctly after low-pass filtering (Kwon & Legge, 2012), suggesting that global, coarse information may also facilitate the recognition of letters or words.

In addition to spatial frequency, color and luminance contrast can also influence reading. For example, Chase et al. (2003) found that skilled readers performed better after removing low frequencies (i.e., red) from the light spectrum, which are thought to suppress activity in the M pathway. In addition, the M pathway may have advantages for attention allocation in reading as compared to the P pathway, as suggested by performance benefits in word recognition tasks that place more demands on the early attention selection of visual features (Omtzigt & Hendriks, 2004; Pammer et al., 2006).

Several studies have investigated whether M information facilitates visual word recognition in the fovea by using a masked repetition priming paradigm. For example, Boden and Giaschi (2009) used spatially low-pass filtered (low cutoff at 2 cycles per degree [cpd]) and high-pass filtered (high cutoff at 8 cpd) stimuli and found that neither low nor high spatial frequency information alone was sufficient to prime lexical decisions. However, when they adjusted the cutoff frequencies (high cutoff to 3.5 cpd and low cutoff to 4.6 cpd), both HSF and LSF information induced equivalent priming effects, suggesting that while LSF information can prime lexical decisions, it is unlikely to play a unique role in word recognition.

In masked priming studies with event-related potentials (ERPs), the vOT regions have been found to be associated with N1 and N250 components (e.g., Holcomb & Grainger, 2007). Given the important role of vOT regions in the framework of predictive coding, this suggests that masked priming could be a suitable paradigm to test M pathway contributions to visual word recognition. In a masked priming experiment with ERPs, Winsler et al. (2017) manipulated the prime's spatial frequency (standard full-spectrum prime, HSF prime, or LSF prime). In the standard and HSF conditions, repetition effects were significant for both the N250 and N400 components, but not for the N1 component. However, LSF primes (supposedly via the M pathway) elicited a delayed N1-like effect; therefore, the role of the M pathway in visual word recognition remains unclear. Recently, Huang et al. (2022) tested the role of the M pathway in visual word recognition in Chinese by manipulating spatial frequency and luminance contrast. They used Chinese two-character words and presented them character-by-character to induce readers' expectations for the second character, enabling top-down processing, supposedly mediated via the M pathway. By combining ERP recordings with a masked repetition priming paradigm, spatial frequency primes that biased toward M or P pathways similarly increased the N1 repetition effect for repeated stimuli compared to unrelated ones. Additionally, when manipulating the bias by luminance contrast, both M-biased and P-biased primes increased repetition effects in N1 and reduced repetition effects in N250. Thus, in line with previous studies (Boden & Giaschi, 2009; Winsler et al., 2017), Huang et al. (2022) did not find distinct contributions of the M and P pathways to visual word recognition; therefore, both pathways may facilitate early visual processing in word reading to similar extents.

Although the evidence from foveal presentation of words does not support stronger facilitation of word recognition by the M as compared with the P pathway, it is possible that differences in facilitation may occur in parafoveal vision, where M cells are more abundant than P cells. For example, Thomson (2007) presented words and nonwords, both foveally and parafoveally, on a red background to inhibit the M pathway. The red background slowed down lexical decisions and led to a greater number of errors for parafoveally presented items, whereas it had little impact on items presented in the fovea, which is dominated by P cells.

However, not all studies found evidence of M-pathway-based facilitation of parafoveal letter identification. For example, Chung et al. (2002) presented spatially filtered lowercase letters to the fovea and parafovea of three observers. While the spatial-frequency attributes for letter recognition were essentially the same in both central and parafoveal vision, the peak sensitivity of the spatial-tuning functions occurred at a higher spatial frequency in the fovea than in the periphery (specifically at 5° and 10° in the lower visual field). Similar findings were also obtained in text reading by Chung and Tjan (2009).

Since most studies have used behavioral measures to investigate the contribution of the M pathway to parafoveal reading, its neural correlates are unknown. The current study aimed to fill this gap by providing neural evidence for the roles of the M and P pathways in visual word recognition by manipulating spatial frequencies (Experiment 1) and luminance contrasts (Experiment 2) in the parafovea and by co-registering EEG and eye movements (Dimigen et al., 2011). Combining EEG and eye tracking provides both temporal and spatial information, as eye tracking can tell us where observers fixate their gaze, and EEG registers when and how the brain responds to visual input, providing precise information about the different cognitive processes occurring during the reading of specific words. In addition, this technique allows readers to move their eyes freely and to read naturally. The EEG signals that are time locked to fixation onsets are known as fixation-related potentials (FRPs). This method has been frequently combined with the boundary paradigm (e.g., Dimigen et al., 2012) where an invisible boundary is embedded between a word *n* and a subsequent word *n* + 1 in the text. Prior to the eye gaze crossing this boundary, different words can be presented as the (parafoveal) preview for word *n* + 1; this preview can be either identical to or different from the actual target that is presented once the readers' eyes have crossed the boundary. The boundary paradigm has most often been used in natural reading scenarios, typically involving sentences as materials, but it also works with word lists (e.g., Dimigen et al., 2012; Kennedy et al., 2002; Kornrumpf et al., 2016) or single words (Rayner et al., 1980).

Preview effects are among the most commonly observed phenomena in eye tracking studies utilizing the boundary paradigm. These effects refer to faster processing of a target when its preview is related, as compared to when it is unrelated to the later fixated word. Eye tracking research has demonstrated that in alphabetic languages, readers can extract orthographic information (Balota et al., 1985; Drieghe et al., 2005; Inhoff & Tousman, 1990; Johnson et al., 2007; White et al., 2008; Williams et al., 2006) and phonological information (Ashby et al., 2006; Chace et al., 2005; Miellet & Sparrow, 2004; Pollatsek et al., 1992). Semantic information can be also accessed in alphabetic languages (Andrews & Veldre, 2019; Antúnez et al., 2022; Hohenstein et al., 2010; Hohenstein & Kliegl, 2014; Milligan et al., 2025; Schotter, 2013, 2018; Schotter et al., 2015; Schotter & Fennell, 2019; Schotter & Jia, 2016; Veldre & Andrews, 2016a, 2016b, 2017, 2018a, 2018b). In Chinese, readers can extract both orthographic (Tsai et al., 2004; Yan et al., 2009; Yu et al., 2019; Zhou et al., 2018) and semantic information (Li et al., 2024; Wang et al., 2016; Yan et al., 2009, 2012; Yang et al., 2012).

More recently, studies combining EEG and eye tracking have found a reduced negativity (termed “preview positivity”) in FRPs following valid compared to invalid parafoveal previews (obtained while fixating on word *n*) in a time window between 200 and 280 ms after fixating on word *n* + 1, which is largest over the OT scalp sites (e.g., Degno et al., 2019; Dimigen et al., 2012; Kornrumpf et al., 2016; Niefind & Dimigen, 2016). The time windows and scalp distributions resemble the effects of masked priming on the N250 component (e.g., Holcomb & Grainger, 2007; Huang et al., 2024). Because the amplitudes for valid previews are less negative (or more positive) than those for invalid previews, the neural mechanism may be interpreted as facilitation due to repetition (Dimigen et al., 2012). Importantly, this facilitation effect appears to require substantial parafoveal processing, as the preview positivity has been shown to occur only for high-frequency previews during sentence reading (Milligan et al., 2023). The preview positivity has been observed in both word list reading (Dimigen et al., 2012; Niefind & Dimigen, 2016) and sentence reading (Degno et al., 2019; Dimigen & Ehinger, 2021).

In addition, although less investigated, there are also preview effects on the earlier N1 component in visual word processing where valid previews elicited larger negativities compared to invalid previews (Dimigen et al., 2012; Dimigen & Ehinger, 2021; Li et al., 2015; López-Peréz et al., 2016; but see also Kornrumpf et al., 2016; Niefind & Dimigen, 2016). This N1 effect typically occurs around 170 ms and is most pronounced over OT regions. Compared to the preview positivity, N1 effects are usually smaller and less consistent. Effects with similar timing, direction and scalp distribution have been reported in masked repetition priming studies (e.g., Chauncey et al., 2008; Huang et al., 2024). The N1 component has been shown to be more sensitive to printed words compared to symbols (Maurer et al., 2005, 2008), and may reflect the mapping of visual features to location-specific letter positions in alphabetic languages, as suggested by masked priming studies (Grainger & Holcomb, 2009).

In the present study, we aimed to investigate whether there is M facilitation during visual word recognition by presenting Chinese characters parafoveally in a boundary paradigm. To activate M and P pathways separately, we manipulated the preview content via spatial frequency (Experiment 1) and luminance contrast (Experiment 2). FRPs were recorded to investigate the neural correlates of the eye-movement effects. Our FRP condition was similar to that in a previous study (Huang et al., 2022) except that, here, we additionally biased the preview stimuli toward the M versus P pathways rather than just showing identical vs. unrelated words as previews.

As in a previous masked priming study (Huang et al., 2022), we used Chinese two-character words as materials, which helped the readers to form expectations about the upcoming character. In Chinese two-character words, each character can serve as a morpheme and the first character can activate the entire word (Taft & Zhu, 1995, 1997) as well as the family words of the morpheme (Wang & Liu, 2020) and hence prime the whole compound word.

An interaction between the preview effect (valid/identical vs. invalid/unrelated preview) and spatial frequencies/luminance contrast would indicate distinct roles for spatial frequencies/luminance contrasts and hence for the M versus P pathways. In general, we expected an attenuation of the N250 (i.e., preview positivity) and a larger N1 for target words preceded by valid previews as compared to invalid previews for all conditions, including the standard condition (with unaltered text). For M- and P-bias manipulations, we expected larger N250 preview effects for M-biased (LSF/heteroluminant) than for P-biased (HSF/isoluminant) previews, in line with M facilitation of early neural processes of word recognition. In addition, we expected similar patterns in eye movement data, where all three types of previews should show preview effects, but these effects should be stronger for M-biased previews (i.e., shorter fixation durations) than for P-biased previews.

## MATERIALS AND METHODS

### Participants

Twenty-four native Mandarin-speaking Chinese participants (14 females, 10 males; mean age = 23.25 yr, *SD* = 2.83) took part in both experiments, which were conducted during the same session. All participants reported normal or corrected-to-normal visual acuity and normal color vision and confirmed they were not suffering from dyslexia or ADHD. They were all right-handed as determined by the Chinese Handedness Questionnaire (Li, 1983). Written informed consent was obtained prior to the experiment. All participants were reimbursed with 50 HKD (approximately 7 USD) per hour. The study was approved by the CUHK Clinical Research Ethics Committee.

### Materials

Stimuli were identical to those employed in a previous study (Huang et al., 2022). Forty pairs of two-character words were selected from the SUBTLEX-CH database of Chinese word and character frequencies (Cai & Brysbaert, 2010). Among these word pairs, the first character was the same in both words (for example, 搏斗 – 搏击; fight vs. boxing). The second character of one word of a pair could serve as the invalid (parafoveal) preview for the other word; for example, in the word 搏斗 (fight), the character 斗 (fight) served as a valid preview, whereas the character 密 (thick), taken from a word not in the pair served as invalid preview. We used this manipulation to help the reader form an expectation for the upcoming character (see also Huang et al., 2022). In Chinese two-character words, both characters can be morphemes, and morphemes activate the entire word, since compound words are stored as a unit (Taft & Zhu, 1995, 1997). In addition, the character in the first position may also prime the corresponding compound words (Myers et al., 2004). Thus, once the first character is presented, it may activate the words within the morpheme family (Wang & Liu, 2020). The two words of each pair were closely matched in word frequency, word contextual diversity (Adelman et al., 2006), character frequency, character contextual diversity, number of strokes, and number of logographemes in the second character. For each word, there was no shared radical. All 80 two-character words (40 pairs) were presented together with valid and invalid previews in three preview conditions (M-biased, P-biased, and standard previews), resulting in 480 critical trials. An additional 72 target words were randomly selected from the 480 critical trials and added to the list as probes, for which participants needed to name the second character of the word. This increased the number of trials in each of the two experiments to 552.

**Figure 1.**
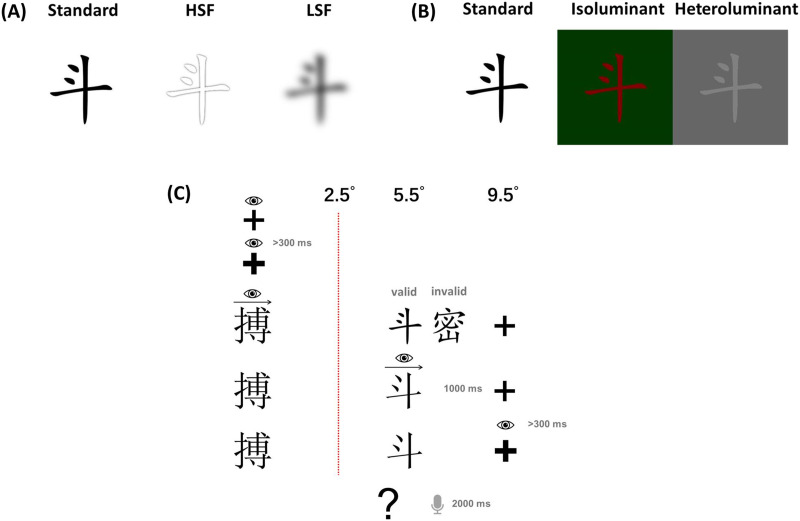
Examples of previews. (A) Experiment 1. (B) Experiment 2. (C) A schematic illustration of a typical trial with valid previews and invalid previews. The invisible boundary is marked by a red dashed line at a visual angle of 2.5°, and the eye symbol represents the current fixation. Note the font change from the valid preview condition (top line) to the fixation of this character (middle line). The visual angle for the preview character is 5.5°, and the right fixation cross is located at a visual angle of 9.5° from the first character. Each line represents the content that participants could see on the screen (gray content was not included). Trials began with a fixation check, during which participants were required to fixate for at least 300 ms before the fixation cross turned bold. This first bold fixation cross signaled that participants could begin reading the words from left to right. The preview was either identical to or different from the target character. The question mark appeared only in occasional trials. When the reader shifted their gaze from the target character to the right fixation cross, that cross also turned bold, indicating the end of the trial (without probes). HSF = high spatial frequencies, LSF = low spatial frequencies.

Characters were presented in black on a white background (except for the M and P-biased manipulation in Experiment 2). Both, the first character and the target stimulus were presented in a Songti font. To reduce visual overlap between preview and target stimuli, previews were presented in a Kaiti font. Stimuli were viewed from a distance of about 90 cm; hence, each character subtended horizontal and vertical visual angles of 1.91°. In Experiment 1, the preview characters were spatially high-pass filtered at 8 cpd (P biased) or low-pass filtered at 2 cpd (M biased). In Experiment 2, achromatic, heteroluminant stimuli were used as M-biased previews; P-biased previews were chromatic with the character and presented on an isoluminant background (red characters shown on a green background with the same luminance, as shown in Figure 1B). The mean Michelson contrast of heteroluminant stimuli was 20% (Green et al., 2009). In addition, unaltered standard preview characters were included in both experiments to replicate established preview effects.

### Procedure

Participants were seated in a comfortable chair, viewing an LCD monitor (24 in. BenQ ZOWIE XL2411K; resolution: 1,920 × 1,080 pixels; vertical refresh rate: 144 Hz) in a sound-attenuated, darkened room. Each experiment comprised six blocks, with two blocks each of standard, P-biased, and M-biased preview conditions. The experiments began with a short practice block, followed by one of the six experimental blocks. Participants performed a naming task in which they were to pronounce the character whenever the target character was followed by a question mark (probe). Participants could rest during breaks between blocks. The six experimental blocks in each experiment were counterbalanced by using a Latin square design such that every target appeared in both the valid and invalid conditions and in each spatial frequency/luminance contrast condition. The order of items within each block was pseudorandomized. The order of the two experiments was counterbalanced as well.

As shown in Figure 1C, each trial began with a fixation cross in the middle of the screen, on which participants needed to fixate for at least 300 ms; otherwise, the trial was skipped. Then, the first character, preview, and a fixation cross to the right were presented together. Participants were instructed to read the words from left to right at their own pace. An invisible boundary was placed at an eccentricity of 2.5° to the first character (determined by a timing test for two different visual angles, ranging from 2° to 2.5°, in 0.5° increments, to make sure that the display change could be triggered with minimal error) to the right of fixation (also determined by a timing test for five different visual angles, ranging from 4° to 6°, in 0.5° increments). The first character was presented in Songti font (a standard Chinese serif font) at the center-left of the screen. The preview character was presented in Kaiti font (a Chinese script-style font) at a visual angle of 5.5° to the right of fixation, and the right fixation cross appeared at 9.5° of visual angle from the center fixation. Once the participants' gaze crossed the boundary, the preview was replaced by the target character (now in Songti font), with the first character and a fixation cross to the right still presented. Once the reader had finished reading the target character and had shifted their gaze to the right fixation cross for at least 300 ms, the fixation cross turned bold. The participants' saccade toward the right fixation cross was required in order to measure fixation duration on the target characters. A minimum fixation duration on the right fixation cross of 0.3 s marked the end of the trial for trials without probes (84% of the trials). In 16% of trials with probes, a blank screen (1 s) was immediately followed by a question mark for 2 s, indicating that participants should pronounce the second character of the word. The responses were audio recorded and manually scored by the experimenter.

Display change awareness was assessed in a structured interview after both experiments had been completed. Participants were first asked whether they had noticed “anything strange about the visual display of the text” (White et al., 2005). If they answered “no,” they were informed that changes had taken place and asked again whether they had noticed any changes (as in Dimigen et al., 2012). If they had, participants were asked to estimate (1) in which experiment they believed there had been more changes; (2) the number of changes perceived per experiment; and (3) the percentage of trials that had included a change per experiment. Due to the simplified setting of the current reading task, we expected that many participants would be aware of the display changes, as there were always font changes between previews and targets. Importantly, however, a previous study had found N250 preview effects regardless of the reader's awareness of display changes (Dimigen et al., 2012).

### EEG Recordings

The EEG was recorded from 64 Ag/AgCl scalp electrodes mounted in a textile cap at positions based on the 10–10 system (Chatrian et al., 1985) and referenced online to CPz. For recording the electrooculogram (EOG) two electrodes were placed on the outer canthus of each eye and one EOG electrode was placed on the infraorbital ridge of the left eye. Signals were amplified with an EEGO amplifier system (Advanced Neuro Technology, Enschede, Netherlands) using a band-pass of 0.01–70 Hz and sampled at 1000 Hz. Impedances were kept below 20 kΩ by using conductive gel.

### Eye Movement Recordings

Eye movements were recorded binocularly using a desktop-mounted Eyelink 1000 Plus eye tracking system (SR Research) at a sampling rate of 1000 Hz. The head position was stabilized via a chin rest. A nine-point calibration was conducted at the start of the experiment, followed by a one-point drift correction check before each trial. Additional recalibrations were performed whenever a check failed. A calibration was accepted whenever the average error was < 0.5° and the maximum error was < 0.99°.

### Co-registration of Eye Movements and EEG Signal

The co-registration of eye movements and EEG was achieved by sending shared trigger pulses from the presentation PC (running Presentation software, Neurobehavioral Systems Inc., Albany, CA) to the EEG and eye tracking computer on each trial through the parallel port. This allowed for accurate offline synchronization of eye movements and EEG signals via the EYE-EEG extension of EEGLAB (Dimigen et al., 2011). After synchronization, the temporal offset between the shared trigger pulses in both recordings exceeded 1 ms only rarely.

### Preprocessing of Eye Movement Data

We analyzed three eye tracking measures for the second character of each word: first fixation durations (FFD; the duration of the first fixation on the target character), single fixation durations (SFD; fixation duration when a character only received one first-pass fixation), and gaze durations (GD; the sum of fixations during the first-pass reading of a character). Fixations were determined by the Data Viewer software with default settings (SR Research). Only fixations that occurred during first-pass reading in correct trials were included in the analysis. Specifically, fixations within the area of interest were excluded if the display change had occurred too late (i.e., when the display change took more than 10 ms after fixation onset on the target character) or too early (i.e., when the display changed before the eye moved across the boundary). We also removed fixations with FFD < 60 ms or > 600 ms and fixations with GD > 800 ms and fixations on target characters that were compromised by a blink. In addition, we removed occasional trials in which participants had moved their eyes from the first character directly to the right fixation cross without fixating the target (*n* = 464, or 3.50% in Experiment 1; *n* = 432, or 3.26% in Experiment 2).

### EEG Preprocessing

Offline, the EEG data were digitally band-pass filtered between 0.1 Hz and 30 Hz (–6 dB/octave), using the default eegfiltnew function of EEGLAB 2021.0 (Delorme & Makeig, 2004) toolbox for MATLAB (Version 2018b), and recalculated to an average common reference (Lehmann & Skrandies, 1980). Using the Infomax algorithm, independent component analysis (ICA) was performed in order to identify ocular artifacts following the implementation in the EYE-EEG extension. ICA components that shared temporal covariance with eye movements greater than 1.1 were removed from the data as oculomotor components (Dimigen, 2020; Plöchl et al., 2012). The EEG signals were segmented from 300 ms prior to 700 ms after fixation onset. Each epoch was baseline-corrected by subtracting the channel mean in the 200 ms interval before fixation onset on the target character. Trials with amplitudes exceeding ±100 *μ*V in any channel were automatically excluded. In addition, we removed trials in which participants had moved their gaze from the first character directly to the right fixation cross (*n* = 21, or 0.16% in Experiment 1; *n* = 45, or 0.34% in Experiment 2). After eye movement and FRP preprocessing, a total of 10,573 observations for the target character remained in Experiment 1, and 10,263 in Experiment 2. FRPs were then averaged per condition and electrode within participants (see Table 1 for final trial numbers). A further post hoc power analysis conducted using PANGEA (Version 0.2; Westfall, 2015) indicated that with around 60 items per condition and 24 participants, the current study design had 80% to detect a small to medium effect size (*d* = 0.32) for the interaction of condition and preview. This represents the minimum detectable effect size for our study design.

**Table 1.** Mean trial numbers after preprocessing (*SD*s in parentheses).

	Standard invalid	Standard valid	P biased invalid	P biased valid	M biased invalid	M biased valid
Experiment 1	56.88 (8.33)	56.96 (9.04)	60.04 (8.47)	58.79 (7.35)	59.54 (8.41)	58.40 (8.39)
Experiment 2	56.42 (13.82)	52.40 (11.66)	55.25 (12.68)	54.04 (11.46)	51.71 (9.83)	53.33 (14.14)

### Data Analysis

#### Eye movement statistical analyses

Eye movement data were analyzed with linear mixed-effects models within the R environment for statistical computing (Version 4.2.3; R Core Team, 2023). We used the lmer function from the lme4 package (Version 1.1.37; Bates et al., 2015) on log-transformed FFD, SFD, and GD. The within-subject factors preview (valid vs. invalid) and type (M biased vs. P biased) were coded as fixed factors. Participants and items were specified as crossed random effects, with both random intercepts and random slopes (Barr et al., 2013). We always started with the maximal models that included the full maximum random effects structure (including slopes). However, slopes were removed whenever the model failed to converge (indicating overparametrization). The *p* values were estimated using the lmerTest package with the default Satterthwaite's method for degrees of freedom and *t* statistics (Version 3.1.3; Kuznetsova et al., 2017). We first ran a linear mixed effect model on the standard conditions to test whether we could replicate typical preview effects. To directly test whether there is M facilitation over P processing in eye movements, we included only M- and P-biased conditions in each experiment with factor preview to test whether the preview effect was larger with M-biased than with P-biased previews.

#### FRP analysis

Separate FRPs were calculated for the six types of target conditions in each experiment. We used the same strategy as Huang et al. (2022), first comparing targets in the valid and invalid preview conditions with standard stimuli to identify time windows with preview effects, and then testing within these time windows whether preview effects differed between M- and P-biased stimuli. To identify the time windows of preview effects in the standard stimuli, we ran point-to-point topographic analyses of variance (TANOVA; Koenig et al., 2011) on non-normalized (raw) scalp maps, comparing target FRPs following valid and invalid previews for the standard conditions. The TANOVA results were corrected for multiple comparisons using global duration statistics (Koenig et al., 2011). Based on the TANOVA results, we selected the time windows in which preview effects were significant (*p* < 0.05). Because we were mainly interested in preview effects corresponding to the N1 and N250 effects, we focused on effects within the first 400 ms for FRPs time locked to the fixation onset of the target words. We then applied the time windows to M- and P-biased conditions to average amplitudes in regions of interest (ROI). Since the N1 effects and preview positivity are distributed mainly in the OT scalp regions, we selected this area as the ROI (left OT: PO9/PO7, right OT: PO8/PO10) with an additional factor, laterality (left vs. right).

We employed Bayesian linear mixed-effects models to investigate the relationship between FRP amplitudes and experimental factors. The models were implemented using the brms package in R (Version 2.22.0; Bürkner, 2017), which provides an interface to Stan for Bayesian inference (RStan Version 2.32.6; Stan Version 2.32.2; Stan Development Team, 2024). In all models, vague priors were used for intercepts and slopes (i.e., normal distribution with *μ* = 0, *σ* = 2). Mixed-effects models utilize all available data points, thereby reducing the risk of biased effect size estimates compared to traditional approaches that aggregate or exclude data.

The strength of evidence for our hypotheses was evaluated using Bayes factors (BF) with reference to Jeffreys' (1939) classification scheme, as updated by Wagenmakers et al. (2018). We adopted BF > 3 as the threshold for moderate evidence favoring the alternative hypothesis, with BF > 10 indicating strong evidence. Conversely, BF < 1/3 provided moderate evidence for the null hypothesis. In addition to Bayes factors, we report posterior mean estimates (in *μ*V) with 95% credible intervals, following recommendations by Makowski et al. (2019). Convergence was assessed using standard diagnostics: at least 2,000 bulk and tail effective sample sizes for each parameter estimate, and R̂ ≤ 1.01, indicating successful convergence across chains.

Separate models were fitted for each experimental condition to address specific research questions. To compare M-biased and P-biased conditions, we fitted a full factorial model including type (M biased vs. P biased), preview (valid vs. invalid), laterality (left vs. right), and all their interactions:

FRPs ~ target_repetition + Preview × Type × Laterality + (1 + Laterality | Subject) + (1 | Item).

For the standard condition, we fitted a reduced model excluding the Type factor:

FRPs ~ target_repetition + Preview × Laterality + (1 + Laterality | Subject) + (1 | Item).

We employed a maximal random effects structure with random intercepts for subjects, items, and target sequences, and by-subject random slopes for laterality effects. This specification accounts for individual differences in lateralization while controlling for dependencies arising from repeated measurements across participants and stimuli (Barr et al., 2013). Models were fitted using Gaussian likelihood, four Markov chain Monte Carlo chains, no thinning, 4,000 iterations per chain (2,000 warm-up), yielding 8,000 posterior samples for inference after warm-up.

## RESULTS

### Behavior Results

Naming accuracy for the targets in the occasional probe trials was very high in both Experiment 1 (99.5%, *SE* = 0.01, range: 95.8%–100%) and Experiment 2 (98.9%, *SE* = 0.01, range: 95.8%–100%).

### Change Awareness Detection

As expected, all participants were aware of the display changes in the experiments; eleven participants reported more changes in Experiment 2 (luminance contrast), and seven participants reported more changes in Experiment 1 (spatial frequency). The remaining six participants estimated the frequency of changes as being similar for the two experiments. On average, participants estimated that they had noticed 159 changes in Experiment 1, which amounts to 61% of the trials in the experiment (actual number: 50%); for Experiment 2 this estimate was 165 changes, accounting for 60% of the total trials (actual number: 50%). In summary, participants were largely aware of the saccade-contingent preview manipulation, but to a similar extent for both experiments.

### Eye Movements

The mean fixation times for each condition are shown in Table 2.

**Table 2.** Means and standard errors of the fixation time (in ms) on the target character.

	Condition	SFD	FFD	GD
Experiment 1	S-valid	238 (12)	231 (9)	250 (13)
	S-invalid	257 (14)	244 (11)	259 (14)
	S-preview effect	19 (7)	13 (4)	9 (4)
	P-valid	224 (10)	219 (8)	234 (11)
	P-invalid	253 (12)	241 (10)	257 (13)
	P-preview effect	30 (7)	22 (4)	23 (4)
	M-valid	226 (10)	219 (7)	236 (11)
	M-invalid	250 (12)	239 (9)	254 (12)
	M-preview effect	24 (5)	20 (4)	19 (4)
Experiment 2	S-valid	241 (10)	228 (8)	242 (9)
	S-invalid	264 (11)	248 (9)	268 (11)
	S-preview effect	23 (6)	20 (4)	26 (5)
	P-valid	237 (13)	227 (7)	242 (8)
	P-invalid	261 (13)	249 (9)	265 (11)
	P-preview effect	24 (6)	22 (4)	22.9 (4)
	M-valid	234 (13)	221 (8)	235 (10)
	M-invalid	251 (13)	239 (10)	260 (13)
	M-preview effect	17 (8)	19 (4)	25 (5)

*Note*. Means and standard errors have been calculated per subject. Preview effects were calculated as valid minus invalid previews. SFD = single fixation duration; FFD = first fixation duration; GD = gaze duration; S = standard condition; P = P-biased condition; M = M-biased condition.

#### Experiment 1: Spatial frequency manipulation

We replicated the typical patterns of preview effects for the standard condition, with invalid previews eliciting significantly longer fixation durations in all eye movement measures (see Table 3). On average, the size of this preview effect was 19 ms in SFD, 13 ms in FFD, and 9 ms in GD (see Table 2). The analysis of differences between M- and P-biased previews showed a significant main effect of preview in all measures. However, the interaction of type and preview was not significant in any of the measures.

**Table 3.** Fixed effect estimates from the linear mixed-effects models on the eye movement data.

		First fixation duration				Single fixation duration				Gaze duration			
	Factor	*b*	*SE*	*t*	Sign.	*b*	*SE*	*t*	Sign.	*b*	*SE*	*t*	Sign.
Experiment 1	M vs. P												
	(Intercept)	5.41	0.04	157.83	< 0.001***	5.50	0.04	135.70	< 0.001***	5.46	0.04	130.53	< 0.001***
	Preview	−0.08	0.01	−8.89	< 0.001***	−0.09	0.01	−8.53	< 0.001***	−0.08	0.01	−7.90	< 0.001***
	Type	0.01	0.01	0.56	0.57	0.00	0.01	0.39	0.70	0.00	0.01	0.36	0.72
	Preview × Type	−0.01	0.01	−0.73	0.47	−0.01	0.01	−1.14	0.26	−0.01	0.01	−0.90	0.37
	Standard												
	(Intercept)	5.44	0.04	153.05	< 0.001***	5.53	0.04	134.10	< 0.001***	5.50	0.04	139.38	< 0.001***
	Preview	−0.09	0.01	−6.34	< 0.001***	−0.09	0.02	−4.70	< 0.001***	−0.11	0.01	−7.35	< 0.001***
Experiment 2
	M vs. P												
	(Intercept)	5.39	0.03	160.16	< 0.001***	5.52	0.04	129.98	< 0.001***	5.48	0.04	142.01	< 0.001***
	Preview	−0.07	0.01	−7.87	< 0.001***	−0.08	0.01	−6.67	< 0.001***	−0.08	0.01	−8.11	< 0.001***
	Type	0.02	0.01	1.65	0.10	0.03	0.01	2.98	0.003*	−0.02	0.01	1.70	0.091
	Preview × Type	0.00	0.01	−0.13	0.90	6.73	6.82	0.99	0.39	0.00	0.01	0.02	0.99
	Standard												
	(Intercept)	5.44	0.04	153.05	< 0.001***	5.53	0.04	134.10	< 0.001***	5.50	0.04	139.38	< 0.001***
	Preview	−0.09	0.01	−6.34	< 0.001***	−0.09	0.02	−4.70	< 0.001***	−0.11	0.01	−7.35	< 0.001***

*Note*. Sign. = significance.

*p < 0.05, **p < 0.01, ***p < 0.001.

#### Experiment 2: Luminance contrast manipulation

Similar to Experiment 1, the standard condition in Experiment 2 replicated the typical and significant preview effects in all three eye movement measures (see Table 3; SFD: a difference of 23 ms; FFD: 20 ms; GD: 26 ms).

When comparing M-biased and P-biased conditions, significant preview effects were found in all three eye movement measures. Additionally, the P-biased condition yielded longer fixation durations in SFD compared to the M-biased condition (but not in FFD and GD). Consistent with Experiment 1, the interaction between type and preview was not significant in any of the measures, suggesting indistinguishable preview effects in M- and P-biased conditions.

### FRP Results

**Figure 2.**
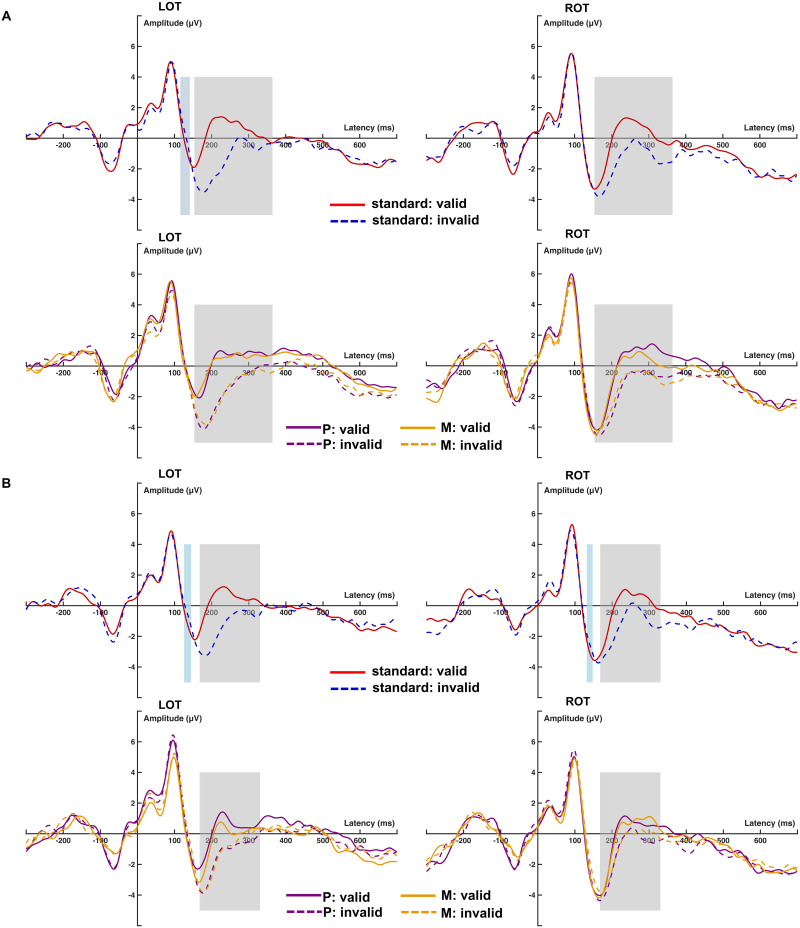
Comparison of target fixation-related potential (FRP) waveforms as a function of preview validity and preview type (standard, magnocellular [M] biased, parvocellular [P] biased). Left and right side of figure show left (LOT) and right (ROT) occipitotemporal regions of interest (ROIs), respectively. (A) Experiment 1. (B) Experiment 2. The top in each panel shows FRPs to targets that had received valid and invalid previews in standard conditions, while the bottom superimposes M-biased and P-biased conditions. The gray rectangles indicate the time windows analyzed. Paired *t* tests were conducted to compare the repeated and unrelated conditions for each standard stimulus. The light blue regions indicate the time windows where significant differences were found between conditions for each ROI (see more details in Discussion). For Experiment 1, 116–141 ms was identified in LOT. For Experiment 2, 126–144 ms was identified in LOT, and 132–147 ms was identified in ROT.

Figure 2 shows the grand-average FRPs, time locked to the first fixation on the target word *n* + 1. Visual inspection of the target FRP waveforms at OT electrodes revealed a biphasic muscle spike potential around time zero (Keren et al., 2010), followed by a dominant occipital P1–N1 complex. This complex consisted of the P1 component, peaking around 100 ms after fixation, and a negative peak around 150 ms, resembling the N1 component in ERPs. After the P1–N1 transition, the FRPs of the standard condition started to diverge between valid and invalid previews during the N1 offset. Based on previous studies (Dimigen et al., 2012), the difference between the invalid and valid preview conditions reflects the preview positivity, or N250 component.

#### Experiment 1: Spatial frequency manipulation

**Figure 3.**
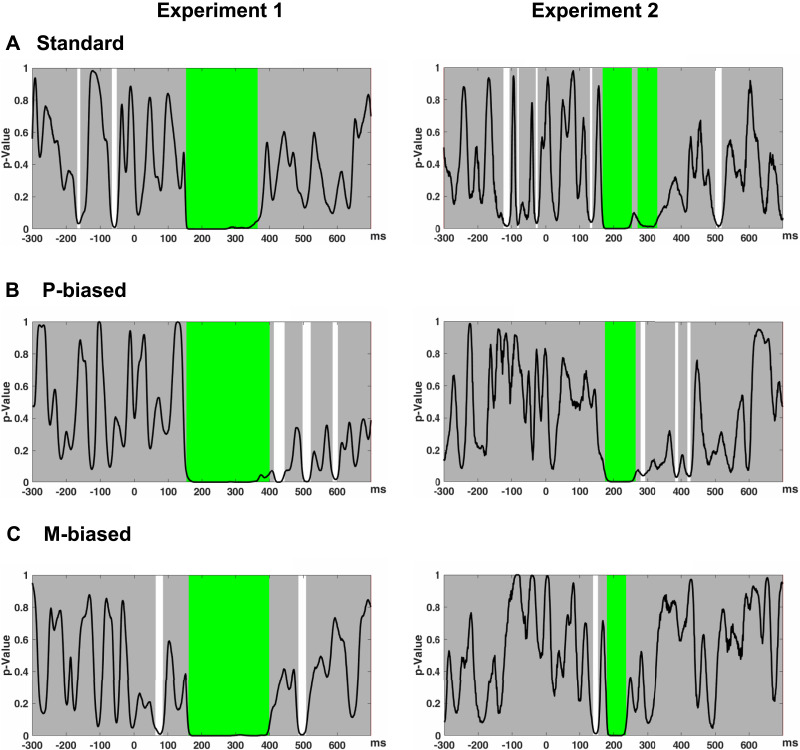
Results of sample-by-sample topographic analyses of variance (TANOVAs) with global duration statistics (marked in green) in Experiment 1 (left) and Experiment 2 (right). (A) Standard condition. (B) P-biased condition. (C) M-biased condition. Each plot visualizes the *p* values (*y*-axis) for the comparison between the mean event-related potential (ERP) maps of each factor level (valid vs. invalid) for every time point (in ms, on the *x*-axis). Gray areas mark nonsignificant time points, while the white areas mark periods of significant differences between ERP maps of different factor levels. Multiple comparisons were corrected for, using global duration statistics, and the duration thresholds were then applied to the TANOVA plots, where periods longer than the estimated duration threshold are marked in green. For the standard condition, the duration threshold was identified as 46 ms for Experiment 1 and 38 ms for Experiment 2. For the P-biased condition, the duration threshold was identified as 45 ms for Experiment 1 and 39 ms for Experiment 2. For the M-biased condition, the duration threshold was identified as 44 ms for Experiment 1 and 37 ms for Experiment 2.

TANOVA comparing valid and invalid targets within the standard condition revealed only one significant time window after fixation onset (see Figure 3A). This window lasted from 154 to 364 ms, consistent with a preview effect on the N250 component in the waveforms. However, no earlier time windows were identified, indicating that the early N1 preview effect may not be robust, at least in the standard condition.

For the standard condition, we observed extreme evidence for the preview effect (Preview: *β* = *–*0.77 [–0.92, –0.62], BF_10_ = 1.99 × 10^10^), with invalid previews having larger negativity compared to valid previews. The evidence for lateralization was inconclusive (Laterality: *β* = 0.21 [0.00, 0.42], BF_10_ = 0.397). The interaction of preview and hemisphere showed strong evidence for the null hypothesis (Preview × Laterality: *β* = *–*0.07 [–0.22, 0.07], BF_10_ = 0.060), suggesting a bilateral distribution for the preview effect. The repetition order of target showed strong evidence for the null hypothesis (*β* = 0.03 [–0.03, 0.09], BF_10_ = 0.021), indicating that the order in which participants experienced target stimuli had minimal influence on the results.

The analysis of the M- versus P-biased conditions showed moderate evidence for the null hypothesis (Type: *β* = −0.18 [−0.48, 0.12], BF_10_ = 0.15), indicating similar amplitudes between M and P conditions. The main effect of preview showed extreme evidence (Preview: *β* = −0.87 [−1.02, −0.72], BF_10_ = 2.44 × 10^9^), with invalid previews being more negative than valid previews. The interaction between type and preview showed moderate evidence for the null hypothesis (Type × Preview: *β* = 0.15 [−0.07, 0.37], BF_10_ = 0.142), suggesting similar preview effects for both P- and M-biased previews. The main effect of laterality showed moderate evidence for the alternative hypothesis, with the left hemisphere having larger activation than the right hemisphere (Laterality: *β* = 0.27 [0.09, 0.45], BF_10_ = 4.28). The type of preview did not interact with lateralization, with strong evidence for the null hypothesis (Type × Laterality: *β* = 0.04 [−0.16, 0.24], BF_10_ = 0.057). The preview effect did not show a reliable hemisphere difference, with strong evidence for the null hypothesis (Preview × Laterality: *β* = −0.05 [−0.20, 0.09], BF_10_ = 0.051). The three-way interaction of type, preview, and laterality showed moderate evidence for the null hypothesis (*β* = −0.13 [−0.34, 0.07], BF_10_ = 0.122). The repetition order of targets did not influence the amplitudes, although the evidence for the null hypothesis was inconclusive (*β* = 0.06 [0.02, 0.09], BF_10_ = 0.526).

**Figure 4.**
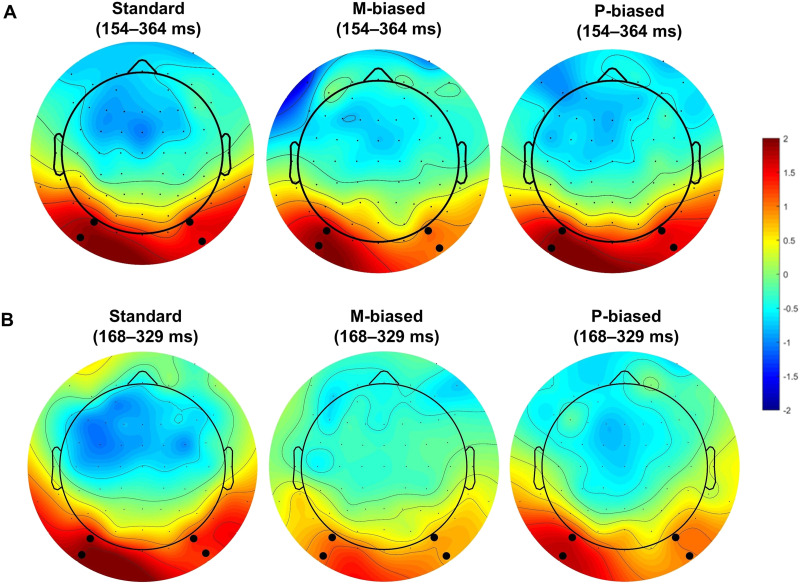
Topographies for the preview effect (valid minus invalid) in FRPs according to preview type. (A) Experiment 1, 154–364 ms window. (B) Experiment 2, 168–329 ms window.

To test whether the results at the ROIs reflected the effects across the entire ERP maps, TANOVA (not normalized) on average amplitudes in the N1 time window was computed with factor type (HSF vs. LSF) and preview (valid vs. invalid). The results showed a significant preview effect (preview, *p* < 0.001) that did not differ between HSF and LSF conditions (Preview × Type, *p* = 0.83). In addition, the FRP maps did not differ between HSF and LSF conditions irrespective of preview relation (Type, *p* = 0.53). The distribution of the preview effect is illustrated in the topography maps in Figure 4A.

To further test whether the time windows of LSF and HSF for the preview effect were different from those in the standard condition, point-to-point TANOVA comparing target FRPs following valid previews to those following invalid previews for LSF and HSF was conducted separately (see Figure 4B and C). For both conditions, a large time window was identified with a preview effect, and this time window overlapped with the one identified in the standard condition (LSF: 162–397 ms; HSF: 152–397 ms). In addition, in the HSF condition, several later time windows beyond 400 ms after fixation onset were identified (409–445 ms, 497–520 ms, and 584–603 ms). In the LSF condition, an early time window of 63–83 ms and a late time window of 482–509 ms were identified, but these did not survive the correction for multiple comparisons.

#### Experiment 2: Luminance contrast manipulation

TANOVA in the standard condition revealed two larger time windows from 168 to 252 ms and 272 to 329 ms with significant preview effects. As the two time windows were separated by only a short interval with marginally significant *p* values < 0.1, we combined them for further analysis, resulting in one large time window from 168 to 329 ms, similar to Experiment 1. In addition, a short early time window from 127 to 137 ms was identified, which, however, did not survive corrections for multiple comparison. Therefore, only the time window of 168 to 329 ms was analyzed, which presumably reflects the N250 component.

Similar to Experiment 1, we first tested within the 168 to 329 ms window, whether the preview effect in the standard condition was lateralized. For the standard condition, we observed extreme evidence for the preview effect (Preview: *β* = −0.91 [−1.08, −0.74], BF_10_ = 4.65 × 10^7^), with amplitudes being more negative to invalid previews compared to valid previews. The amplitudes were similar in both hemispheres, with moderate evidence for the null hypothesis (Laterality: *β* = 0.13 [−0.09, 0.35], BF_10_ = 0.115). The preview effect showed a bilateral symmetric distribution, with moderate evidence for the null hypothesis (Preview × Laterality: *β* = −0.15 [−0.30, 0.00], BF_10_ = 0.243). The repetition order of targets showed strong evidence for the null hypothesis (*β* = 0.03 [−0.02, 0.09], BF_10_ = 0.025).

To address the key question of whether heteroluminant (M-biased) previews produced a greater preview effect than isoluminant (P-biased) previews, we ran the Bayesian linear mixed models at OT regions on factors type (valid vs. invalid), preview, and laterality. Results of the analysis of M- versus P-biased conditions showed strong evidence for the null hypothesis for the main effect of factor Type (*β* = 0.06 [−0.23, 0.35], BF_10_ = 0.079). There was extreme evidence for a main effect of preview (Preview: *β* = −0.68 [−0.85, −0.51], BF_10_ = 2.92 × 10^6^), indicating that invalid previews were more negative than valid previews. However, the preview effects did not differ between types, with moderate evidence for the null hypothesis (Preview × Type: *β* = 0.20 [−0.04, 0.45], BF_10_ = 0.222). The main effect of laterality showed strong evidence for the null hypothesis (Laterality: *β* = 0.02 [−0.18, 0.22], BF_10_ = 0.052), suggesting a bilateral distribution. The interaction of type with laterality showed strong evidence for the null hypothesis (Type × Laterality: *β* = −0.08 [−0.31, 0.15], BF_10_ = 0.070). The preview effect was similar in both hemispheres, but the evidence was inconclusive for the null hypothesis (Preview × Laterality: *β* = −0.18 [−0.34, –0.02], BF_10_ = 0.490). The three-way interaction of type, preview, and laterality showed moderate evidence for the null hypothesis (*β* = 0.10 [−0.13, 0.33], BF_10_ = 0.080). The repetition order of target showed strong evidence for the null hypothesis (*β* = 0.02 [−0.02, 0.06], BF_10_ = 0.017).

To further test whether the ROI results reflected the effects across the FRP maps, TANOVA on average amplitudes in the N250 time window across all electrodes were calculated. TANOVA revealed a significant preview effect (preview, *p* = 0.0004), which did not differ between heteroluminant and isoluminant conditions (Preview *×* Type, *p* = 0.923). In addition, FRP maps did not differ between heteroluminant and isoluminant conditions as a main effect of type (*p* = 0.26).

As in Experiment 1, we tested whether the time windows of the preview effects in the isoluminant and heteroluminant conditions differed from the standard condition. Point-to-point TANOVAs comparing FRPs following valid previews with those following invalid previews were conducted separately for each of the two conditions (see Figure 3, right panels). The results revealed some small additional time windows which did not survive multiple comparison corrections. For both conditions, we identified time windows that overlapped with our previous analysis (168–329 ms): isoluminant stimuli showed effects from 171 to 262 ms, while heteroluminant stimuli showed effects from 179 to 235 ms. In addition, for the isoluminant condition, a short time window of 281 to 293 ms was identified. Similar to TANOVA on the standard condition, we think this short time window still reflects the preview positivity, as this effect has the same direction and similar scalp distribution of the N250 effect. Moreover, two short time windows of 382–392 ms and 419–428 ms were identified, with the largest activation in the central-parietal regions. Together with waveforms that show invalid previews tending to be more negative compared to valid ones, we consider these two time windows to possibly reflect the N400 component. For the heteroluminant condition, only a time window from 138 to 152 ms was revealed, which may reflect the N1 component.

## DISCUSSION

The present study used FRPs and eye tracking to investigate whether parafoveal M information facilitates the early neural processes underlying visual word recognition more strongly than parafoveal parvocellular information. Furthermore, as in a previous study with masked repetition priming (Huang et al., 2022), we induced expectations about the upcoming character, by using Chinese two-character words presented character by character; hence, participants could form an expectation about the full two-character word on the basis of the first character. Most importantly, in addition to normal standard previews (with full spatial frequencies), we presented P-biased and M-biased parafoveal previews. This was achieved by separately manipulating the preview's spatial frequency (Experiment 1) and luminance versus color contrast (Experiment 2). Our results show robust parafoveal preview effects in all measures of eye movement behavior. Furthermore, in FRPs, we observed a consistent reduction of the N250 component following valid as compared with invalid previews. This “preview positivity” was found in the standard, P-biased, and M-biased conditions, suggesting that our manipulation of parafoveal preview was successful. Importantly, however, our Bayesian linear mixed-effects model analysis revealed that no single pathway contributed significantly more than the others. We discuss these findings below.

### N250 Preview Effects

FRP amplitudes in the standard conditions were more positive (less negative) following valid previews compared to invalid previews within the time window of about 150 to 300 ms after fixation onset on the target character in both experiments. This N250 preview effect was largest over OT regions. In terms of latency and topography, this effect likely corresponds to the “preview positivity,” which has been hypothesized to reflect a partial activation of abstract orthographic and phonological representations (Dimigen et al., 2012). Compared to the N250 effect of masked priming in ERPs observed by Huang et al. (2022) using the same materials, the preview positivity in the current findings started earlier already during the N1 offset and lasted longer. In addition, the size of the effects in the present study was larger than those in Huang et al. (2022). It is possible that this is due to the active nature of the current task requiring oculomotor movements, as contrasted with the masked priming paradigm of our previous study where eye movements were to be avoided (Huang et al., 2024). Active saccade behavior has been shown to change the deployment of attention and increase the size of preview effects (Kornrumpf et al., 2016; Niefind & Dimigen, 2016).

### No N1 or N400 Preview Effect

Contrary to our hypothesis, we observed no robust early effects in the standard condition of both experiments, although some transient effects were noted in the P1–N1 transition before multiple comparison correction (see light blue shadings in Figure 2). To enhance statistical power, we combined the standard condition across experiments to test whether we could observe any early effects. Consistent with the TANOVA results for the standard condition in each experiment, apart from the one (160–380 ms) corresponding to the N250 component, we only observed a short time window of 127–145 ms, which was identified but failed to survive global duration statistics (41 ms) before 400 ms after fixation onset. In addition, we plotted the significant time windows for each experiment in the corresponding ROIs in Figure 2. Therefore, we primarily relied on the TANOVA results from the standard condition in each experiment for the data analyses.

This observation contrasts with the previous masked priming study using very similar stimuli (Huang et al., 2022), where N1 repetition effects were robust. In FRP studies, preview effects on N1 are less frequently reported (e.g., Dimigen et al., 2012). The P1–N1 transition is associated with visual categorization processes sensitive to word orthography (Proverbio et al., 2004) and modulated by spatial attention (Miniussi et al., 2002; Valdes-Sosa et al., 1998). The absence of clear P1–N1 preview effects in our study may be due to differences in paradigms. In the boundary paradigm, unlike in masked repetition priming, readers move their eyes freely. This could lead to central saccadic suppression inhibiting early visual processing of postsaccadic input (Temereanca et al., 2012). However, these potential costs are likely offset by increased parafoveal preprocessing (i.e., N250) at the saccade goal during active reading.

Compared to previous studies using masked priming (Huang et al., 2022; Winsler et al., 2017), neither an N400 nor a P300 was found as preview effects in the present study. The reason could be that in a boundary paradigm, the preprocessing of parafoveal information is increased (Kornrumpf et al., 2016). Therefore, it is possible that readers process the visual inputs more efficiently during the N250 interval in the current situation, as the N250 preview effects lasted longer.

### No Unique Contribution of the M Pathway to the N250 Preview Effect

The main goal of the present study was to test whether the N250 preview effects were mediated by the fast M pathway. In this sense, we expected that the M-biased conditions would show the N250 preview effect also seen in the standard condition, but that the P-biased condition would not; in other words, we expected the N250 preview effect to be larger in the M than in the P condition. However, this hypothesis was not supported. The Bayesian analysis revealed that when manipulating spatial frequency, the results actually supported the null hypothesis, suggesting that the two pathways play a comparable role in visual word recognition.

One traditional view suggests that the M pathway plays a critical role in reading (see the magnocellular theory of developmental dyslexia; Stein, 2001). Contrary to the traditional view of strictly parallel M and P pathways, recent evidence reveals extensive interactions and convergence between these systems at multiple stages of visual processing (Freud et al., 2017; Nassi & Callaway, 2009). Developmental and clinical evidence support this integrated model, showing that M–P interactions are essential for normal visual function and that pathway damage affects both systems due to their interactions (Ajina et al., 2015; Ellis et al., 2021; Skottun, 2015). Also, multimodal neuroimaging and computational modeling converge on a revised understanding of M and P pathways' functions as an integrated network with extensive cross talk (Benson & Winawer, 2018; Mitchell et al., 2014). Evidence from developmental dyslexia involves inefficient M–P coactivation rather than isolated M deficits (Ciavarelli et al., 2021). Visual word recognition is complex, requiring readers to not only identify the word shape but also to recognize subtle differences. Thus, the M pathway may help with making fast global inferences, while the P pathway can support identifying fine-grained details, allowing readers to use both word shapes and detailed features. Our study did not show unique contributions of one pathway relative to the other; thus, according to the magnocellular-parvocellular coactivation hypothesis, readers may integrate both fine-grained and global shape information based on both pathways. Therefore, modern reading models should incorporate dynamic M–P integration rather than assuming independent parallel processes. Visual word recognition may emerge from coordinated pathway interactions at multiple processing stages (Grainger & Holcomb, 2009).

### Preview Effects in Eye Movements

We replicated typical preview effects in fixation times for all conditions, including M- and P-biased previews, showing that both fine-grained and coarse word shape information facilitate word recognition. Importantly, the preview effects in fixation times were robust but not modulated by the type of preview (M vs. P biased), indicating that there was no major difference in the facilitation of parafoveal visual word recognition provided by either pathway. Therefore, the eye movement data strongly support the null effect for the interaction, indicating that M- and P-biased information similarly contribute to character recognition at the behavioral level.

### Variability of M- and P-Biased Manipulation

The precise selection of spatial frequencies may be critical for observing path-specific modulations. Unlike previous studies manipulating spatial frequency, our approach preserved more LSF information. Winsler et al. (2017) used 15.2 and 3.7 cpd for HSF and LSF, respectively, while Boden and Giaschi (2009) employed 4.6 and 3.5 cpd. Notably, Boden and Giaschi (2009), when filtering at 2 and 8 cpd for LSF and HSF, found no priming effects for either LSF or HSF information in a lexical decision task. Despite using a similar manipulation, our results demonstrated that both M-biased and P-biased stimuli led to neural facilitation and preview benefits in eye movements. This suggests that the specific method used to measure priming or preview effects is crucial in assessing the effectiveness of stimulus manipulation.

### Conclusion

To summarize, we used combined eye tracking/EEG to show the neural facilitation of visual orthographic processing by valid parafoveal information, as evidenced by a robust preview positivity. A unique role of the M pathway for preview effects in visual word recognition was not found, which was also supported by a Bayesian analysis that provided evidence for the null hypothesis regarding spatially filtered M- versus P-biased stimuli. Instead, both M and P pathways seem to contribute to early processes of visual word recognition, indicating that efficient reading relies on both pathways.

## Funding Information

Urs Maurer, Hong Kong Government (https://dx.doi.org/10.13039/501100017649), Award ID: RGC-GRF 14616418.

## Author Contributions

**Xin Huang:** Conceptualization; Data curation; Formal analysis; Investigation; Writing – original draft; Writing – review & editing. **Brian W. L. Wong:** Conceptualization; Investigation; Writing – review & editing. **Werner Sommer:** Investigation; Writing – review & editing. **Olaf Dimigen:** Investigation; Writing – review & editing. **Urs Maurer:** Funding acquisition; Conceptualization; Supervision; Writing – review & editing.

## Data and Code Availability Statements

All data and code are publicly available via the Open Science Framework at https://osf.io/abrpc/.

## TECHNICAL TERMS

Magnocellular pathway: The retinal ganglion cells have large (magno) cell bodies. The magnocellular pathway carries information about large, fast things (low spatial frequency, high temporal frequency) and is colorblind.
Parvocellular pathway: The retinal ganglion cells have small (parvo) cell bodies. The parvocellular pathway carries information about small, slow, colorful things (high spatial frequency, low temporal frequency).
Fixation-related potentials (FRPs): Averaged brain potentials aligned to fixation onsets.
Contextual diversity: The proportion of texts in which a given word occurs.
Michelson contrast: The absolute difference of fore- and background luminance values divided by their sum.
